# Challenges and Strategic Solutions to Guarantee Last Mile Reach for an Indian TB Patient’s Nikshay Poshan Yojana; A Conditional Cash Transfer Scheme: Comment on "Does Direct Benefit Transfer Improve Outcomes Among People With Tuberculosis? – A Mixed-Methods Study on the Need for a Review of the Cash Transfer Policy in India"

**DOI:** 10.34172/ijhpm.2023.7668

**Published:** 2023-04-19

**Authors:** Harsh Shah

**Affiliations:** Department of Public Health Science, Indian Institute of Public Health - Gandhinagar, Gujarat, India

**Keywords:** Tuberculosis, Nikshay Poshan Yojna, TB Control Program, Conditional Cash Transfer, Health System Strengthening, India

## Abstract

India has put efforts into the prevention and control of tuberculosis (TB) for more than 50 years. Nikshay Poshan Yojna (NPY) is one of the schemes of conditional cash transfers (CCTs) by the Government of India. The CCT schemes mostly address the demand side constraints. Governments could use this in developing nations as a tool to divert financial resources toward societal development. In India, NPY is more directed toward providing monetary support for a nutritional diet and reducing the catastrophic expenditure of TB patients. Several studies highlighted challenges in implementing cash transfer schemes and provided different operational models. A country like India should address the challenges with defined strategies to ensure its last-mile reach. A present commentary discussing challenges and possible solutions that policy-makers can adapt and set up a support structure to ensure that supportive actions are implemented in response to patient and system side issues.

## Background

 Dave and Rupani highlight an important issue from the Indian context of the program implementation framework.^1^ India has put efforts into the prevention and control of tuberculosis (TB) for more than 50 years. Yet, TB is one of the major causes of high morbidity and mortality. The National TB Elimination Program, formerly known as the Revised National TB Control Program, oversees the TB control efforts and has recently been updated with new diagnostic tools, treatment plans, and public health action plans. India still has a lot of “missing” cases each year that are not reported and may not be covered.^2^ According to the “National Strategic Plan for Tuberculosis Elimination 2017–2025,” the Government of India intends to eradicate TB by 2025. New initiatives supported, monitored, and managed at all levels are essential to accomplish such a monumental undertaking.

 The main causes of the rising number of TB cases are the expanding population, poor diet, a lack of a yearly healthcare budget, unsanitary living conditions, drug addictions, etc. A healthy diet plays a crucial part in the fight against TB.^3,4^ In reality, most TB patients are either too weak or too poor to afford a diet high in nutrients. Additionally, the loss of daily pay owing to illness exacerbates the patients’ precarious financial situation.^5-7^ Nikshay Poshan Yojna (NPY) is one of the schemes of conditional cash transfer (CCT) by the Government of India.^8^ There are various CCT schemes in the health system for patients. NPY is more directed toward providing monetary support for a nutritional diet and reducing the catastrophic expenditure of TB patients enrolled under the National TB Elimination Program.^9^

## Conditional Cash Transfer Schemes

 The basic idea behind CCTs was developed in Latin American nations, mostly as a reaction to the macroeconomic crisis of the 1990s, during which it was believed that demand for social services like education and health from poorer households had sharply decreased. Such programmes often try to shorten periods of extreme poverty while long-term safeguarding the development of human potential.^10^ When a household or individual satisfies certain requirements, such as attending health clinics or using government health services, they give disadvantaged households cash directly. These programmes signify a change in the government’s strategy from one that focuses on the supply side to one that is demand-driven. Several studies highlighted the critical operational aspects of cash transfer interventions and various TB-specific, TB-inclusive and TB-sensitive implementation models.^11^ As CCT schemes need to be tailored according to the socioeconomic nuances of a region; it requires state and local governments to play a more active role in financing and monitoring such schemes.

## Field Challenges in Implementation of the NikshayPoshanYojna

 The incentive of NPY is transferred directly to Aadhar (a unique identification number given to every citizen) linked bank account of the TB patients through a specifically designed Public Finance Management System. To receive the benefits of NPY, confirmed diagnosed TB patients have to submit their Asdhar card and bank account details to the concerned health staff for registration. The key feature of this CCT is that it is not linked with any conditional terms related to treatment adherence or following specific criteria related to the TB program. Also, it is for all TB patients irrespective of their age, gender, socioeconomic strata, site of disease (Pulmonary or Extra-pulmonary), type of case (New or Relapse), drug susceptibility (first line/ second line drug-sensitive or resistant) and duration of the treatment regimen. There may be challenges in the implementation of these CCT schemes; especially NPY, as below:

1. Registration of the patients: As many patients are from marginalized populations – intravenous drug users, destitute, etc, migrants, construction workers, etc and their identification proofs and Aadhar card details may not be available to them. Also, using a bank account or opening a zero-balance bank account is still a concern in developing countries.
2. Delay in disbursement and receipt due to lengthy approval process: The process involved from registration to disbursement takes multiple steps through the different levels of authority in the healthcare system. Though the direct bank transfer to beneficiaries’ account reduce the leakages but still the delays in the timely receipt of payment were one of the major issues, and that hinders the prime objective of nutritional support.
3. Non-conditional financial assistance: Apart from being a TB patient on treatment, there are no other set conditions for NPY. The financial aid may lead to malpractices, such as the problem of substance (alcohol, tobacco, drugs, etc) abuse among TB cases looms large, and so providing financial assistance to them may encourage them to continue these social malpractices, thus directly affecting the treatment outcomes. Also, from the system side, monitoring the usage of received monetary benefits is not in place, which remains a major gap in the health system for all CCTs in India.
4. Perception of being stigmatized and non completion of treatment: The scheme has set eligibility criteria based on confirmation of disease and treatment initiation. From the patients’ perspective, the patients may be seen as contagious, and they may be reluctant to continue with their course of treatment out of fear of being “found out” and consequently stigmatised. The desired effect of NPY—the high coverage of treatment completion—cannot be attained.

## Addressing Demand Side Barriers

 The evidence from different nations shows that the programmes incentivise households to change their behaviour toward nationally recognised social goals when supply limitations are not severe.^11,12^ The CCT schemes were originally conceptualised to minimise the negative externalities that downturn the community’s socioeconomic profile and to attract the marginalised poor households to participate in government schemes. These schemes can potentially shift human behaviour and financial resources in the desired direction over time. Still, deploying interventions properly to achieve the desired results might be difficult. Governments could use this in developing nations as a tool to divert financial resources toward societal development. National and state governments must take a more active role in funding and overseeing CCT schemes since they must be customised to the socioeconomic specifics of a region. Dave and Rupani reported a delay in reaching the benefits to the target beneficiaries and the purpose not served among the poor society. Despite an increase in inflation since the CCTs were implemented in India, the distribution amount has not altered or increased. Various studies from India suggested that the overall implementation of these schemes has improved service delivery.^13^ The following areas require urgent attention, and the policy-makers of National TB programs implementing CCT schemes should reinforce them (Table 1).

**Table 1:** Strategies and Supportive Activities to Address the Demand Side Barriers for Conditional Cash Transfers

**Strategy**	**Key Activities **
Influencing public opinion and local action through public health communication	Customised strategies to tackle contributory factors such as TB underdiagnosis, including stigma, poor disease knowledge and perception of healthcare services on the patient level.
Estimation of the budget requirement for disbursement to the beneficiaries	Periodic need assessment of the minimum financial requirements for the beneficiaries and establishing the projection model of budget requirements over the years.
Identifying the expenditure pattern by the community	Assessing the dietary patterns, culture and customs, per capita income, treatment cost and receipt of services from government and private hospitals, and total catastrophic expenditure pattern on health. Creating a system to get feedback on the expenditure pattern of monetary support received from various CCTs.
Enriching the awareness about the DBT schemes and program interventions	Community mobilisation through customised advocacy and communication strategy for the geography-specific community about DBT schemes and payment mechanism.
Energising action to address the social determinants of health	Prioritising actions towards social determinants of health, including poverty, economic, social and gender inequity, and malnutrition with strong political will and leadership.

Abbreviations: TB, tuberculosis; CCTs, conditional cash transfers; DBT, direct benefit transfer.

## Addressing System Side Barriers

 In developing nations, the situation of the health system across the program interventions plays a major role. Significant issues like system inaccessibility, resource unavailability and poor quality of services are still hindering the delivery of universal health coverage. The demand side CCT schemes will raise awareness somehow, but system strengthening requires several key interventions. First and foremost, a nation like India should concentrate on unblocking domestic financing sources and directing cost-effective initiatives throughout the health system. Spending on health infrastructure should be enhanced to establish connections between the community and the health system. This includes both facility-based interventions and community-based interventions. As the Lancet Commission report predicts we will be in until at least 2045, the focus should continue to be on raising investment from all sources as long as we are still in the pre-elimination period.^14^ The report also suggested that the countries should enhance their absorption capacity to spend the allocated funds for the National TB Programs. Simultaneously, the monitoring and evaluation capacity of the CCTs schemes needs to be addressed systematically to ensure the desired impact not only on health parameters but also on the socioeconomic condition of the beneficiaries. The policy-makers may prioritize the following strategies to ensure the last mile reach of the CCTs schemes (Table 2).

**Table 2:** Strategies and Supportive Activities to Address the System Side Barriers for Conditional Cash Transfers

**Strategy**	**Key Activities **
Addressing the social determinants of the health	Developing an institutional framework to address social inequalities and limited access to healthcare by involving all government departments and civil societies with strong political will.
Health system strengthening through the universal healthcare approach	Availing of the resources in response to the need and ensuring an uninterrupted supply of human resources, machines, materials and money to deliver the health services up to the last mile.
Strong financial disbursement system to reduce delay	Development of training cascade for the staff involved in CCTs starts from registration to disbursement and creating a reward mechanism based on performance for the staff.
Establishing the surveillance sites for assessing the cost-effectiveness of the CCTs	Creating geographic-specific, marginalized population-specific, social strata-specific surveillance sites for collecting impact data of the CCTs.
Periodic evaluation of the schemes based on achievement of desired targets	Periodic evaluation of the schemes for the set conditions needs to be conducted and based on results and a series of consultations; revision of conditions, if required, should be implemented.

Abbreviation: CCTs, conditional cash transfers.

 It is well known that the high catastrophic cost of TB care throughout treatment affects households in India, including non-medical expenses such as transportation, daily wage loss, etc.^15^ A CCT programme like NPY should demand far greater attention from policy-makers and more comprehensive data on its effects on TB epidemiology. The national TB programme should set up a support structure to ensure that supportive actions are implemented in response to patient and system side issues.

## Ethical issues

 Not applicable.

## Competing interests

 Author declares that he has no competing interests.
